# Previs d'Anatomic Transcendentale appliquée a la Physiologie

**Published:** 1845-01

**Authors:** 


### PRECIS D'ANATOMIE TRANSCENDANTE, APPLIQUÉE A LA PHYSIOLOGIE. Par *M. E. R. Serres*, Membre de l'Institut, Professeur au Museum d'Histoire Naturelle, &c. Tome Iier. 8vo. pp. 270. Paris.

WE hope that none of our readers will allow themselves to be scared from the perusal of the following pages by the epithet that is here applied to the Sciences of Anatomy and Physiology. The term, " transcendental," is a very unfortunate one; for, without conveying any distinct intimation of its meaning, it at once suggests, in the present day, the idea of some abstruse mysticism about matters that are beyond the reach of common minds. True it is that of late years it has rarely been used, except in reference to the metaphysical doctrines of Kant and other German writers; but the word, although rather pedantic, is nevertheless of English growth; for we find it in the works of some of our older authors. Dr. Johnson defines it, in his Dictionary, to be " general," " pervading many particulars," " supereminent;" and *Bailey* explains the expression of *transcendentals* as signifying the " universal conceptions of things." Let us now hear in what sense our author uses the term. " Transcendental Anatomy," he says, " is that branch of physical science, which treats of the mode in which animal bodies are developed, and of the successive changes or phases that they undergo during this process; and which at the same time points out the various analogies that may be traced between these changes in the embryos of the higher animals, and the permanent forms or structures of those that are lower in the Zoological scale."

The field of enquiry thus embraced is a very wide one; for it comprehends not only the perfect and mature organisms of the various orders of animal beings, but also these organisms in the different stages of their evolution and growth—from the period of conception to that of their complete development. The phenomena, therefore, of which it treats, naturally resolve themselves into two great groupes; viz., on the one hand, the transitory forms which the organisms in the higher animals exhibit during their development; and, on the other, the permanent forms of these organisms in the lower animals. As we follow out this curious subject of enquiry, we shall find that there is good reason to believe that the primitive condition of all animal structures is very nearly, if not altogether, the same; and that the chief differences, which they afterwards display, may be traced to the operation of a few simple laws that we shall endeavour in the following pages to point out and explain.

From this brief statement, it will be obvious that the phrase, Transcendental Anatomy, has a meaning not very different from that of Formative or Embryological Anatomy; using the latter expression in its largest and most comprehensive sense,—to denote the study of the evolutionary formation of the different organisms or structures in all animals, and of the various analogies and resemblances that exist between them. Need we add that the study is one of the very highest philosophical interest, and that it therefore behoves every medical man, who has the dignity of his profession at heart, to make himself acquainted with its general bearings and best established phenomena?

The science of Transcendental Anatomy and Physiology is, in a great measure, one of modern creation; for,—although we meet with not a few allusions to its particular doctrines, and in several instances with some very happy conjectures on the subject, in the writings of the old anatomists—it was certainly not, until about the beginning of the present century, that a decidedly successful attempt was made to discover any of those laws, which preside over and regulate the development of animal structures.

This somewhat remarkable retardation of embryological science will be found, we think, to have arisen from the same source of error, that proved so injurious to the advancement of other branches of knowledge, before the discipline of the Inductive Philosophy was rightly understood. Physiologists had always been too ready to frame theories, before they had provided themselves with the proper materials to base and build their theories upon. Thus, in reference to our present subject, it was too generally taken for granted that the embryo of an animal was, from its first appearance, only a mere miniature representation or copy of the being from which it sprang; and hence it came, almost as a necessary consequence, that almost every physiological enquirer concluded that the process of development was nothing more than a gradual evolution and expansion of different parts and structures, that were already existing in the nascent organism. There were indeed, it was confessed by all, some peculiarities in the conformation of the embryo, that it was by no means easy to account for in this way, and which certainly seemed to be at utter variance with the idea of different organs being developed *ab initio* in the exact condition which they afterwards exhibited. For example, the question would naturally propose itself, why were the Testes formed in the loins, and afterwards transmitted through the abdominal rings into the bag of the Scrotum? and what explanation was to be given of the primary formation and subsequent removal by absorption of the *membrana pupillaris* of the eye? On the subject too of Organic Anomalies—or Monstrosities, as they have been rather inaptly called—no rational hypothesis could be even so much as suggested. To refer them to the operation of disease in the Fœtus itself, or to the influence of the maternal mind on her offspring, or to the direct agency of diabolic malevolence, &c. might indeed be a ready way of solving the difficulty to the minds of the vulgar; but no sensible Physiologist could be satisfied that such causes—if we can deign to call them so—should occasion, for example, a Harelip or a cleft palate in one instance, and the absence of the Heart or of the Brain in another.

The whole of the first part of M. *Serres*' work is occupied with an exposition of the two leading

*Theories of Embryotic Development*—the ancient and the modern. According to the one, we are taught to believe in the presence of the different organisms of the body in the embryo, from the very commencement of its being; while the other is based on the idea that there is a series of successive developments and phases of evolution of all its parts, beginning in the most simple forms, and these gradually becoming more and more complicate. The former our author designates "The Doctrine of Pre-existences;" and the latter "The Doctrine of Epigenesis."

He gives a rapid sketch of the writings of physiologists—from the time of Aristotle down to the close of last century—in as far as they bear on this subject, and from this review he shews how almost universally the first of these theories prevailed until the end of last century.

We cannot afford space to follow him through the early part of this enquiry, but shall at once proceed to notice the account which he gives of the labours of our distinguised countryman *Harvey*—premising, however, that our readers must take it for proved that the Doctrine of Pre-existent formations has been shewn to be utterly untenable.

"With that eagle glance," says M. *Serres*, "which characterised all the efforts of his genius, Harvey had made some approaches to a far more correct system of embryological doctrine than was prevalent in his day; and he would probably have anticipated many of the unlooked-for discoveries of modern times in this most interesting branch of anatomical science, had he not stopped short in his enquiries, and commenced to indulge in speculations, instead of continuing persevering to observe and record the phenomena of nature. As he could not see the embryo in the *chalazæ* of the ovum, or the entire chick in the *cicatricula*—assertions that were almost universally believed in his day—he very naturally remarked; *Either it is true, or it is not. If it is, let me see it; but, if it is not, why should we suppose that it must exist? The science of realities is sufficiently tedious and irksome in itself; why then add to it the study of our dreams and the contradictions of our fancies?*" It was thus that this great man at once put a stop to the reception of the fantastic theories which were then in vogue: fortunate would it have been for the interests of Science, as well as for his own reputation, if he had rigidly observed his own precept. But, although (as we shall afterwards see) he, after entering upon the narrow path of truth, subsequently diverged into the broad and beaten track of error, "he,"—again, to use our author's language—"compensated for his mistakes by suggestions and intimations that almost rival in importance his immortal discovery of the Circulation. His memorable saying, *all animals proceed from an ovum,* is the very basis and fundamental position of every rational system of embryology: for certainly no one now denies that an ovum is the universal *matrix* of animal life. And if it be admitted that all animals proceed from a common elementary formation, who does not perceive, in this simple but expressive formula, the germ of the Doctrine of the Primitive Analogy that exists between them? —that doctrine which M. *St. Hilaire* has worked out with so much ingenuity and success;—and is not the physiological enquirer led, at the same time, to begin to suspect that the evolution and development of all animal structures must be determined by the same general laws, and after one common model or type? Harvey's own words clearly shew that he took this view of the subject; for we find him saying, in his great work on Generation, *Quippe in omnibus eodem modo atque ordine procreatur; præsertim in perfectioribus animalibus, atque ipso adeò homine.* As a necessary consequence of this and such-like statements, he clearly foresaw, as we might anticipate, the grand discovery of the present century, viz.: that the embryos of the higher tribes of animals, and of man himself, must, during the progress of their development, pass through conditions which characterise the organisation of animals that are beneath them in the scale of existence. The passage in his *Exercitatio Anatomica de Motu Cordis*, in which he distinctly enunciates this memorable and somewhat startling truth, is too important not to be quoted as it stands in the original: *Sic natura perfecta et divina, nihil faciens frustra, nec cuipiam animali cor addidit ubi non erat opus, neque, priusquam esset ejus opus, fecit; sed iisdem gradibus in formatione cujuscunque animalis, transiens per omnium animalium constitutiones (ut ego dicam), ovum, vermem, fœtum, perfectionem in singulis acquirit. Hæc alibi in fœtus formatione multis observationibus confirmanda sunt.*"

In this brief sentence we recognise the germ, so to speak, of the Epigenetic Doctrine of Embryology, and of some of the most important discoveries which have adorned this branch of science in the present century; for here we have it clearly enunciated that the various organs and structures of the animal do not exist in the primitive *nucleus* or *cicatricula* of the Ovum; but, on the contrary, that they are subsequently and successively developed by the addition, the super-position, and the cohesion of different parts.

So far, therefore, Harvey was altogether on the right way; but, at this very point of his dissertation, we perceive that a spirit of speculation unfortunately begins to be blended with the details of actual observation.

Among other fantasies, he adopted the strange notion that the commencement of existence in the higher animals is to be dated only from the earliest appearance of the pulsations of the heart; and that the anterior development of the Embryo should be considered as appertaining to vegetable rather than to animal life. This utterly unsupported hypothesis led him to entertain the belief that it was from the heart,—the *punctum saliens*—as from a centre, that all the subsequent growth of the young being proceeded. Here was the grand error that he committed; the false step that led him away from the rich prize of discovery that now seems to have been almost within his grasp.

It is singular to find, as we pursue our enquiries, that the really truthful part of Harvey's exposition of Zoogeny was so little attended to by subsequent anatomists. Even Haller—who had examined most attentively for himself many of the phenomena exhibited by the embryo, at an early period of its existence, and was perfectly well aware of the marked difference in many of its parts *then*, and at the time of its mature development—adopted (we might almost say, in spite of his own observations) the generally received doctrine, that the young being is nothing but a mere miniature representation of its ultimate formation, and therefore that the process of development consists in the mere evolution or expansion of originally existing parts.

The absurdities, to which this belief carried him, are worthy of notice only as affording an instructive warning to future physiologists. Without embracing the opinions of Aristotle and Harvey, that the life of the Embryo was merely of a vegetable type or nature before the appearance of the *punctum saliens*—for this opinion had been completely overthrown by the beautiful microscopic researches of Malpighi—he boldly hazarded the extraordinary idea that, although the heart be not visible and even not formed, its impulsive action might nevertheless exist. In short, he believed that there might be a function present without an organ, an acting power without an instrument to work with; and, what is singular, this very supposition led him to interpret the development of the Sanguiferous System in the very inverse order to that which he had discovered by his observations to be the case. So far astray will one wrong step often lead even the most enlightened physiologists.

There were two contemporaries of Haller, whose observations and writings tended not a little to shake the general belief in the Doctrine of Pre-existent Formations, and thus to pave the way for the introduction of the rival creed in Embryology—that of Successive and Consecutive Developments, or, as M. Serres terms it, the *Epigenesis* of animal structures. The first of these was Needham, who examined with great care the mode of development in the Infusory Animalcules which are found in vegetable infusions; and who—without apparently being aware of the ideas of Harvey respecting the transformation of animal beings during their evolution—came to the conclusion that, in some, the progress of development was arrested at one period; in others at a second; and in others at a still more advanced period or stage of their growth. He therefore very properly inferred that the primary germ or ovum of the animal being could not contain any miniature representation of its future framework; but, on the contrary, that there was a progressive addition of new parts or formations until it (the fœtus) was completely and maturely developed. As a corollary deducible from these views, we cannot be much surprised that Needham entertained the belief that animal and vegetable substances are primarily of the same nature, and that, under certain circumstances, they are convertible the one into the other.

Wolf confirmed the accuracy of many of the observations of the Dutch physiologist, and shewed that the different organs of an animal body are evolved in succession; one *after* the other, and even one *from* the other, in the way of secretion. To him we owe the profound remark that "primarily all the parts of an animal are in a fluid and, as it were, an inorganic condition, and that subsequently vessels are developed within it by a peculiar and self-generating action."

After the time of Wolf, nothing important was done in the field of Embryological research, until the beginning of the present century, when the admirable investigations of M. *Geoffroy St. Hilaire* in France, and of *Meckel* in Germany, were made known to the world. These, in connection with the subsequent labours of *Oken, Spix, Baer, Blainville, Serres,* &c. have completely established the foundation, and reared the superstructure of the modern theory of embryology—the most prominent and indeed the essential positions of which are—1, that the embryos of all animals are at first nearly or altogether alike; and 2, that those of the higher orders of animals pass through a series of successive phases of development, each of which has its type or analogue in the permanent configuration of the lower tribes of animal beings.

To understand this theory aright, it is necessary that the reader be first made acquainted with the two great laws of Embryological development, for the clear enunciation and establishment of which, science is mainly indebted to the genius and industry of our distinguished author. These are the laws of the Primary Duality, and of the Centripetal or Excentric formation, of all animal organisms.

By the first of these expressions, M. Serres means to intimate that all animal bodies are primarily developed *bifid* or *dual; i. e.* as consisting of two lateral and similar halves, each one of which is an exact representation of the other. An obvious consequence of this law must be that all the different organs, situated in the median line, will at first consist of two homologous portions—developed apart; but which may, or may not, afterwards unite or coalesce, so as to constitute but one individual organ.

As we proceed in our enquiry, we shall meet with numerous illustrations of this law; but perhaps the best exposition of its truth will be made by tracing the early changes observed during the development of the chick. The following extract from our author may therefore be read with considerable interest:

"The *ovum* in the female before impregnation consists of three fundamental parts—an investing external membrane, the *chorion;* a vesicle called the *proliferous vesicle;* and the *vitellus.* This last part is at first colourless and without any yellow globules. As these form, the Proliferous Vesicle, which is filled with a clear fluid, rises to the surface of the Vitellus, and is maintained in its place there by a disk or ligament, to which the term of *proliferous disk* has been given. The object of the excentric position of the Vesicle seems to be that the seminal fluid or *Zoosperm* of the male may be brought immediately in contact with it—an indispensable condition to all fecundation. The immediate result of this contact is the rupture of the Vesicle, and the escape of its contents,—now impregnated by the Zoospermic agency—upon the capsule of the Vitellus. The first 12 or 15 hours of incubation are employed by nature to isolate the Proliferous Disk from the membrane of the vitellus; the embryo thereby acquiring an area of independent existence (le champ de l'embryon prend ainsi son indépendence). As yet, there is no appearance of any germinating process in the substance of the disk itself, and it continues to be homogeneous throughout. At about the 16th hour of incubation, we perceive the first trace that precedes the development of the organisms. This consists in the appearance, in the axis and about the centre of the disk, of a darkish-coloured line which gradually extends, first upwards and then downwards, to its other extremity. While this is taking place along the median line of the Disk, the edges of its circumference begin to bulge out a little, and they gradually become more and more raised up, until at length there are formed two cells or sacs (*sacci germinativi*), one on each side of the dark-coloured streak in the middle. This therefore may be said to be the first act of development, viz. the division of the proliferous disk into two equal parts—a right and a left one—which are separated from each other by an interval known by the name of the *primitive line.* Embryologists have not been agreed as to what constitutes this line of demarcation; but we feel quite satisfied that in truth it is nothing more than a mere empty space or interval between the two germinating sacs; and therefore that it is neither the *zoosperm* as Prevost and Dumas have imagined, nor the rudiments of the spinal marrow, as Wagner has supposed."

According, then, to our author, the Embryo is to be regarded, at this period of its existence, as consisting of two lateral and similar halves, in each of which the process of development goes on equally and simultaneously. This is the key, so to speak, to that great and important law in Embryology, which is designated by our author as the Law of the Symmetry or Duality of the organism:—it is sometimes called, in compliment to him, the *Lex Serriana*. Our limits forbid us to enter, at any considerable length, into an illustration of this fundamental principle; and we must therefore refer the curious reader to M. Serres' treatise for particulars. Suffice it for our purpose to say that, according to him, (and other high authorities on the subject,) all the viscera and organs of the body consist at first of two distinct lateral halves, equal and similar to each other in all respects, but which may subsequently coalesce and become incorporated together, so that no trace of their primary median division remains.

That the Cerebro-Spinal Axis is developed in this manner is abundantly obvious: the two lateral cords of the spinal marrow are so distinct in the young embryo of Birds and Reptiles, and even of Mammals, that no one thinks of disputing the fact in the present day. In many of the *invertebrata,* this Duality is persistent during life; while in others, the nervous axis becomes single during the process of evolution.

The same thing may be predicated of the Heart, the Liver, and other organs, which in after-life are found to be not only *impar*, i. e. consisting of two unequal halves, but are situated altogether on one side of the body. However startling the assertion may be, there is ample reason to admit that, at first, these parts were all placed in the median line of the body, and that then they consisted of two equal lateral moieties, which subsequently coalesced, and became so incorporated, as to constitute but one undivided mass. The conformation of the Penis in the male, and of the Clitoris in the female, affords a good illustration of this. The lateral halves, of which these organs originally consist, sometimes do not coalesce uniformly and throughout their entire extent; and then we find that a deep rut or groove remains after birth along their upper surface, in the direction of the median line. At other times, the Canal of the Urethra does not close, but continues more or less completely open, along the entire extent of its under surface. The explanation of such irregularities is very simple and obvious: the two lateral halves, of which the parts originally consisted, have not become joined together, as they normally are, so as to form a uniform and continuous whole. In the example now quoted, we have a most convincing illustration of the process of "Dual Formation;" but perhaps not more conclusive than what is afforded by congenital fissures of the lips, palate, and uvula, or by Spina Bifida, or by a bilobed state of the uterus, &c.

The consideration of this elementary position prepares us for the better understanding and the more ready admission of the other fundamental principle in Embryology, which M. Serres has the merit of having first clearly enunciated, and the truth of which he most ingeniously labours to establish by many and varied arguments—we mean the "Centripetal Law," as he terms it, "of Development." By this expression we are to understand that all living organic matter is primarily developed at the surface, and that the process or act of Development goes on in a direction inwards, from the surface towards the centre. If we take the Brain, for example, it is asserted by our author that the periphery of its hemispheres is formed long before the *corpus callosum,* or the *septum lucidum* is even visible. If the truth of the general position be admitted, we have then a very marked feature of distinction between the ancient and modern theories of Embryotomy. As we have already stated, it was the almost universal belief of the old physiologists, that the development of the various parts and organs of an animal body proceeded from the centre to the circumference; and we have seen how Harvey, after having made some important discoveries in the right way, allowed himself afterwards to be diverted from it by his favourite dogma, that life emanated from the *punctum saliens,* as from a starting point. Now the very reverse of all this is at length discovered to be the case, and we are now assured that the circulatory and nervous systems are developed according to the Centripetal Law, *viz,* the blood-vessels at the periphery first, and subsequently the Heart; the nerves first, and subsequently the Brain and Spinal Marrow.

A vast number of the phenomena, exhibited by organic anomalies or Monstrosities, are in strict accordance with this physiological principle or position. The peripheral parts of the body as a whole, as well as of its separate organs, are never found to be awanting when the central parts are developed. For example, we never meet with a monster that is provided with a heart (however rudimentary this may be), but without blood-vessels; or with a Cerebral or Spinal Axis, but without nerves. Again, there is no instance known of one of the cheeks being absent, while the lips were formed; or of a lateral half of the penis being awanting, while the canal of the urethra was entire. And why?—simply because the formative process in every part goes on from the circumference towards the centre; and never in the opposite direction.

Having thus, although very imperfectly, noticed two of the leading elementary laws of Organology—those of the Duality, and of the Centripetal formation of the body—we are now prepared to advance another step in our enquiry; *viz.* to advert to a third general law or position of the science, which it is very necessary to understand, before we can explain many of the phenomena of animal structures. It is called by our author the "Law of the Fragmentary Division *(fractionnement)* of the Organs." The following extract will best convey his meaning.

"Whatever be the organ of the body, whose development we endeavour to follow out, we shall find that it primarily consists of several pieces or divisions; its mature and permanent form being the result of the coalescence and fusion together of these separate parts. The Liver was originally composed of three or four separate portions or lobes; eventually these become so blended together that the lines of junction are scarcely, if at all, recognisable. In the same manner, the Prostate Gland was at first trilobed or quadrilobed. The Kidney in the adult state is a simple uniform organ, whose surface is so smooth that it exhibits not the slightest traces of any previous disjunction; and yet, in the embryonic state of the human being, this viscus, it is well known, has a multilobular formation, as permanently exists in quadrupedal mammalia. The development of the double or compound teeth affords another illustration of the same law. Who would suppose that they consisted at first of several distinct portions or segments? or that each eminence on their exposed surface was originally formed by itself, and apart from the others? Yet this has been discovered to be the case; and we are now assured that, as the number of these eminences varies from two to five, so there may be as many as ten distinct *nuclei* of growth in the formation of a single tooth. It thus appears that the grinders of ruminant animals are in truth only an aggregation of several incisors into one, while those of the carnivora may be regarded as a mere groupe of canine teeth, that have become coalesced together."

Many other instances, illustrative of the same general fact, might be quoted from the development of the osseous system—all tending to prove that parts, which are single and uniform in the adult, are *fractionnés* into several segments during their early formation. Each Vertebra, for example, is found to exhibit four distinct *nuclei* of ossification; the Occipital bone originally consists of eight separate pieces, and the Sphenoidal of no fewer than twelve. M. *Serres* remarks that the original nuclei of separate ossification may often be detected in the adult bone, by the mere circumstance of these points being always of a closer and harder texture than the rest of its substance. The shafts of the long bones are much harder than their extremities; the lateral masses of the vertebræ than the bodies of these bones; the posterior lamina of the occipital bone than its basilar process; and so forth. "The solidescence is therefore," adds he, "a sure guide to discover in the bones of adult animals the original traces of their osteogeny. Such being the case, we can easily perceive the application that might be made of this law to Palæontology or Fossil Anatomy, not only as regards the bones of the vertebrate, but also the solid parts of invertebrate, animals. In the formation of shells, too, and the outer cases of insects and the crustacea, we find that the development commences in those points which are the hardest and the most compact. This is a general rule of Organogeny."

The primary Fragmentary Division of Organs may therefore be received as one of those important general truths of embryological anatomy, which it is the great object of the present work of M. *Serres* to establish; and certainly none is more curious than this, viz: that the organs of the body are more and more decomposed or subdivided into fragmentary segments, in proportion as the embryo is less and less advanced in its development: the earlier the embryo, the greater is the amount of *fractionnement.*

With these facts in view, it will not be difficult to recognize some of the very remarkable features of resemblance that may be traced between the successive transient states, which the Embryos of the higher animals exhibit during their development, and the permanent forms that exist in the lower tribes of animal beings. It may be laid down as a general law that the organs in animal bodies become less and less complex in structure, and more resolved into their elementary constituent forms, in proportion as we descend in the zoological scale; until at length we find them reduced to their most simple and elementary configuration in the lowest members of the series. To illustrate the truth of this statement, let us examine the Comparative Anatomy of the Heart for example. Very complicated in the higher Vertebrata, this organ becomes gradually more and more decomposed—*i. e.*, resolved into its primary elements, so to speak—in Reptiles, Fishes, Crustaceous and Molluscous animals, the Annelides, and Insects. In each of these successive *degradations*, we find that it loses either some part of its component elements, or a portion at least of its muscular apparatus; so that, at length, it becomes nothing more than a straight or slightly-curved canal, whose very muscularity is problematical. Now this is nearly its condition in the early human embryo. When first recognizable, it is not unlike to the normal and permanent structure of the organ which we perceive to exist in worms and insects. The earliest change, that is observed during the process of embryotic development, is the appearance of rudimentary auricles. When these are finally formed, the heart consists of three cavities, viz: a ventricle in the middle, and an imperfect auricle, at some distance, on each side,—an arrangement, it will be perceived, that is very much the same as what exists in the Acephalous Mollusca. Subsequently the two auricles appear to approximate and join each other; and at length these two cavities form but one. Then we have a largely-developed ventricle, and a still larger single auricle—the very structure that is present in the Cephalous (cephalés) Mollusca. What is the next change? The single auricular pouch becomes divided into two compartments by a *septum*—the existence of which, according as it is more or less complete, represents the condition of the organ as we observe it to be in certain Fishes and Reptiles. The last phasis of this "strange eventful history" is when the ventricle itself becomes in its turn bilobular, in the same manner as the single auricle did. At the period when the ventricular septum is not completely closed, have we not an exact representation of the permanent condition of the heart in the Ophidian Reptiles?

The Osseous System also furnishes us with many illustrations of this highly curious and interesting *Law of Concordance between Organogeny and Comparative Anatomy.* The following extract from our author will best serve to convey our meaning:—

"We thus see—and this is a point well worthy of the attention of anatomists—that, whenever an organism is found *fractionné* in the human embryo, we may be assured that it is permanently multiple, or even that it has its component parts detached from each other, in some of the lower animals. The great number of the cranial bones in fishes, when compared with the number of the primary *nuclei* of ossification in the cranium of the early fœtus, affords one of the most remarkable examples of this general law of Organogeny. M. *St. Hilaire* has, with surpassing ingenuity, developed and illustrated this subject. Another instance is met with in the case of the *sternum;* the numerous pieces of which, in several Mammals as well as in Reptiles and Fishes, are perfectly represented by the number of the primary *nuclei* of this bone in the human fœtus, from the second to the fifth month of intra-uterine life. The same holds true with respect to the *os hyoides.* What variety does this osseous apparatus exhibit first in Mammals, and next in Birds, Reptiles and Fishes! * * * * * Again, in the human fœtus, from the third to the fourth month, the *superior maxilla* is found to consist of five distinct pieces, which afterwards coalesce and form a single bone: in the Crocodile, these five pieces remain permanently separate. In respect therefore of this bone, the Crocodile may be regarded as the permanent representative of the human embryo in the third month of its development; and *vice versâ,* the early embryo in this respect may be regarded as a transitory Crocodile, becoming at a future period of its intra-uterine existence a type of a Pachyderm or Ruminant animal, when the incisive portion of the maxillary bone is all that remains distinct and separated from the rest."

A still more curious feature of resemblance between the human embryo and the inferior animals is the unquestionable existence of a caudal prolongation in the former, from the 5th to the 7th week of its development. How would Lord *Monboddo* have rejoiced in spirit, had this discovery been made in his day!

The Visceral or Splanchnic System is no less prolific in examples of the same law of structural concordance than the vascular and osseous systems. The *kidney*, as we have already mentioned, is multilobed in the early fœtus; so it is in the elephant, the cow, &c. The *prostate* gland consists of four lobes in a three-month fœtus; so it does in the horse. The *uterus* is bilobed in a great many of the lower animals; the same arrangement exists in the human embryo. The changes, which the external organs of generation undergo during fœtal life, are perhaps as marvellous as any of the organic metamorphoses to which we have alluded. At a very early period, there appears to be no distinction of sex; subsequently all embryos seem to be females; and at a more advanced period, the female-looking organs appear to be transformed into male ones. All the females, at a certain period of their existence, have the appearance of being hermaphrodites; and all the males exhibit, at some time or another, that of females.

Having next very elaborately pointed out the successive transformations in the structure of the *encephalon*—each of these transformations having its type or analogue in the permanent condition of this organ in the different orders of the Vertebrata, from the Mammal down to the fish—M. Serres prefaces his description of the evolution of the alimentary passages with the following remarks:—

"The essential character of animal life does not reside either in the heart and blood-vessels, in the brain and nerves, or in the genito-urinary organs. An animal being may exist and live without possessing these organisms. Not so however with the nutritive apparatus. Life exists, and can exist, only on this condition—that an absorption of assimilable molecules is continually going on. Hence it follows, on the one hand, that, as we descend in the scale of animal beings, we at length come to those which are reduced to a state of a mere absorbing bag or vesicle; and, on the other hand, that, as we ascend as far back as possible in our embryogenic examinations, we find the rudiments of the future man to be nothing more than a similar vesicle. Thus, the Monad is represented, so to speak, in embryogeny by the *proliferous vesicle* that has been so accurately described by Purkinje; and the Volvox and Proteus by what has been called the *cicatricula ovi*. In the primitive embryo, as in the Polype and Entozoon, the alimentary canal has no anal orifice, nor is there any appearance of a liver or other glandular appendage. It is in the Crustacea, Insects, and the Mollusca, that we first meet with such organs: the same holds true of the embryo, in what may be termed the second stage of its development. In the ulterior metamorphoses or changes, which the digestive apparatus undergoes in the embryo, other and very remarkable analogies with the permanent forms, that are present in different tribes of the lower animals, may be readily pointed out."

If space permitted, we might also allude to the resemblances between the Respiratory organs in the Invertebrata, and those in the embryos of Vertebrate animals. Probably most of our readers are aware that, long before the development of proper *lungs* in the latter, the respiratory apparatus is essentially of the nature of *gills;* and that the young of certain animals of the Reptile tribe are at birth provided with the latter—to be replaced by the former at a future period of life, when the animal passes, for example, from the state of tadpole to that of the frog. Those, who wish to be acquainted with the analogical phenomena in the conformation of the early human embryo, should consult our author's description.

There still remain one or two other topics introduced and commented on in this truly original work, to which we would solicit our readers' attention for a few moments, before drawing our remarks to a close: and first of what M. Serres calls the *Gradation of Tissues in Zoological and Embryological Formations.*

"The primary or elementary tissue," says he, "in the lower Infusoria, as in the embryo during the first stage of its existence, is mere cellular substance, modified in a variety of ways. This is the common basis, so to speak, of the Monad, the Volvox, the Acephalocyst, the Ascidia, and of part of the Echinococcus and Polype. The essential properties of this tissue seem to be—1, a uniformity of function, limited to exhalation and absorption; and, 2, a power of independent existence, when a portion is detached from the rest. If to this tissue be superadded a peripheral system of blood-vessels, we advance one step in the animal scale, and come to part of the Echinodermata. If a muscular system be then added, we reach the Rotifera; and, if all these systems be combined, we arrive at the Helianthoidea. Whenever the muscular tissue is distinctly developed, we find that traces of a nervous apparatus—hitherto so blended and confused with the different parts of the animal body as not to be recognisable—begin to appear; as we observe for the first time in the Annelides, the Mollusca, and perhaps also the Crustacea. Now, a very striking analogy may be traced between these progressive gradations in zoological development and the successive changes that are discoverable in the evolving organisation of the embryo. Before impregnation, the animal is represented by a mere vesicle that consists altogether of cellular membrane enveloping an oleaginous fluid. After impregnation, the *blastoderm*—from which proceed the constituent parts of the embryo—exhibits two membranes; one external and serous, and the other internal and mucous. In a short time, a vascular tissue is found to be interposed between these two; and subsequently a nervous tissue makes its first appearance."

Some light has been unexpectedly thrown on this subject by the results of certain experiments, which M. Serres has recently performed on worms and leeches. "The common earth-worm," he remarks, "is very different, it is well known, in its conformation from the Polype, the Tænia, the Helianthois, or the Arenicola; but, if we follow out the various metamorphoses, which the animal undergoes before it arrives at its complete development, we find that it actually does resemble each of these creatures successively according to its epoch of evolution. Now what is very remarkable is, that a counterproof of these successive advances or elevations of structure is afforded by watching the process which Nature follows in effecting the regeneration of a part, that may have been artificially destroyed. The *first* act of regeneration in the new-formed portion reproduces exactly the structure of an Arenicola; the *second* act (supposing the new-formed has been again destroyed) reproduces that of the Helianthois; while the third and last act degrades or brings the part down to the structure of a Polype.* It is obvious, therefore, that the reproductive

---

* M. Serres mentions that, while conducting these experiments on earthworms, he quite satisfied himself that many of the alleged different species of this and other Invertebrate animals are, in short, but the same species, only at different stages of their development. The most advanced development is the ideal type of the genus; and the least advanced constitutes the last species of it. This remark is, in an especial manner, applicable to the case of the Infusory animalcules, which have too often been most unnecessarily grouped into a great number of different families.

power becomes gradually more and more feeble, just in the same manner as we observe that the reproduction of the organic tissues in the human body become exhausted, in any part, by a frequently-repeated act of regeneration. This may be not unfrequently seen in cases of old ulcers. A first, a second, and even a third cicatrisation gives birth to a new structure that is resistant, and approaches in its characters to the normal condition of the part; but, if this act of reparation be repeated more frequently, the new-formed tissue becomes less and less completely organised : the ulcer is then said to be *atonic,* and under such circumstances it often remains incurable. The same remark is applicable to tuberculous formations, which occur in the lungs and other parts of the body."

He proceeds to point out certain analogies that may be traced between the imperfect or irregular developments—in other words, the Monstrosities —that not unfrequently occur in the human embryo, and the normal structures in some of the lower animals. We must confine our notice of this curious subject to the giving of one short extract.

"These mutilations or privations of organs are incompatible with the continuance of the independent and extra-uterine life of the fœtus; but, although this be quite true, let it not be forgotten that, despite the presence of such imperfections of organisation, the creature was alive while it remained in the womb and was attached to the mother. Nay more, it may already have passed through several stages or phases of existence, and have perhaps more than completed its term of life as an Invertebrate being. Few physiologists seem to be aware that there is in these monstrous or deformed productions a scale, so to speak, of uterine viability (capability of life)—a circumstance of high philosophical importance, as it tends to shew that there is a sort of independent existence on the part of the embryo, during its intra-uterine life. Thus, a fœtus, that is destitute of an extremity only, will continue to live much longer within the womb, and thereby acquire a much higher degree of development, than another in which the heart or the brain is wanting."

In drawing these remarks to a close, we cannot but express a hope that they may excite the curiosity of some of our readers, and induce them to examine, more minutely for themselves, the highly interesting subject of Embryology in connection with that of Comparative Anatomy. No medical man can be said, in the present day, to be duly educated, if he has not acquired a tolerable knowledge of the latter science. How should it be otherwise, when we consider that Physiology can never be studied as it ought to be, without a constant reference to the organisation of all classes of animals, from the simplest Zoophyte up to Man himself? He is but the last link of the chain that extends unbroken through the long series of zoological formations, and every part of which, however dissimilar and unlike one may *seem* to be from another, is constructed upon a simple harmonious and kindred plan. The marvellous discoveries that have been made, of late years, in Embryology, have added not a little to the interest of this subject, and have brought to light many such unexpected analogies and features of resemblance between forms that had been hitherto imagined to be most unlike, as almost to warrant the startling observations of our author, that "Human Organogeny is only a sort of transitionary Comparative Anatomy, as in its turn Comparative Anatomy is only the fixed and permanent condition of the different phases of Human Organogeny;" and that "Animals, in a *genetic* point of view, may be regarded as the permanent embryos of Man."

In contemplating the succession of phases, or stages of development, which the human being is found to exhibit during its evolution within the womb—commencing in the most simple, and terminating in the most complex form, when it reaches its mature and complete formation—are we not almost involuntarily reminded of that most interesting discovery of modern Geology, viz: that just in proportion as we trace back the history of the earth in its physical structure, by examining the older and deeper strata of its formation, so, it would seem, the more simple and uncomplicated were the forms of animal beings then in existence? In the *transition rocks* we meet with the fossil remains of the very lowest tribes only; in the *secondary*, the remains are of animals of a higher order; and it is not until we reach the more recent deposits that the bones of the mammalia are ever found. Is it not possible to trace here a sort of parallelism between the *genesis* of animal beings during the successive epochs of the world's formation, and that of the embryo during its successive gradations of evolutionary development? The thought is but a passing fancy: and as such only we give it. But, whether there be any truth in the conjecture or not, of this we may rest assured, that the more that we search into the hidden workings of Nature, and make ourselves acquainted with her teeming wonders, the more clearly we shall perceive a unity of design and a simplicity of operation pervading all created things, and the more strongly shall we *feel* that they have all been fashioned and ordained, and that they are all upheld and continued, by one Almighty hand. It has been beautifully said that—

The very law which moulds a tear,
And bids it trickle from its source,
That law preserves the Earth a sphere,
And guides the planets in their course.

And so it is with the still greater marvels of the animated world. The same law, that presides over the formation of the humblest creeping thing, directs the development of him who walks the earth

"With port sublime and hopes beyond the skies."

All are alike framed on one simple and universal plan; all are created from a few, and the same simple elements; all perform similar functions; and all at length become resolved into the same component parts. Ought not the study of such a theme as that which we have been glancing at, while it exalts our admiration of the supreme wisdom and power of the Divine Architect, to serve at the same time to humiliate the pride and arrogance of Man? In his body he is but a step removed from the brutes that perish; and, at one period of his existence, he has been but as they. His structure is not more wonderful than theirs; his physical powers are in many respects inferior. It is the spirit within that alone stamps him with pre-eminence, and lifts him so far above every other earthly thing "wherein the breath of life is." Admirably hath the poet expressed the antithetic constitution of his nature—physical as well as moral—when he exclaims—

How poor, how rich! how abject, how august!
How complicate, how wonderful is Man!
* * * * *
An heir of glory! a frail child of dust!
Helpless immortal! Insect infinite!
A worm! a God!

